# PIVKA-II serves as a potential biomarker that complements AFP for the diagnosis of hepatocellular carcinoma

**DOI:** 10.1186/s12885-021-08138-3

**Published:** 2021-04-13

**Authors:** Honglei Feng, Bole Li, Ze Li, Qian Wei, Li Ren

**Affiliations:** 1 Department of Laboratory, Tianjin Medical University Cancer Institute and Hospital, Tianjin’s Clinical Research Center for Cancer, Key Laboratory of Cancer Prevention and Therapy, National Clinical Research Center for Cancer, Huanhuxi Road, Hexi District, Tianjin, 300060 China; 2 Department of Pharmacy, Tianjin Medical University Cancer Institute and Hospital, Tianjin’s Clinical Research Center for Cancer, Key Laboratory of Cancer Prevention and Therapy, National Clinical Research Center for Cancer, Tianjin, China

**Keywords:** Prothrombin induced by vitamin K absence or antagonist-II (PIVKA-II), Hepatocellular carcinoma (HCC), α-Fetoprotein (AFP), Diagnostic biomarkers

## Abstract

**Background:**

Hepatocellular carcinoma (HCC) is one of the most common malignant tumors of the digestive system and has high morbidity and mortality rates. It is essential to search new biomarkers to improve the accuracy of early HCC diagnosis. Therefore, we evaluated the diagnostic value of prothrombin induced by vitamin K deficiency or antagonist- II (PIVKA-II) as a potential biomarker that complements α-fetoprotein (AFP) in HCC by detecting the serum PIVKA-II levels.

**Methods:**

Serum PIVKA-II levels were compared in 168 HCC patients, 150 benign liver disease patients and 153 healthy controls to investigate the PIVKA-II potential to be a HCC biomarker. Receiver operating characteristic curve (ROC) analysis was used to evaluate the value of PIVKA-II in the diagnosis of HCC and its complementary role of AFP. The correlation between serum PIVKA-II levels and clinicopathological characteristics was analyzed to study the value of PIVKA-II in assessing HCC progression and prognosis. Finally, the ability of PIVKA-II in assessing the surgical treatment effects of HCC was studied by comparing the pre- and post-operative serum PIVKA-II levels in 89 HCC patients.

**Results:**

Serum PIVKA-II levels in HCC patients were significantly higher than that in patients with benign liver disease and healthy controls. The PIVKA-II performance in the diagnosing HCC as an individual biomarker was remarkable. The combined detection of PIVKA-II and AFP improved the diagnostic efficiency of HCC. PIVKA-II retained significant diagnosis capabilities for AFP-negative HCC patients. Significant correlations were found between PIVKA-II expression levels and some clinicopathological characteristics, including tumor size, tumor stage, tumor metastasis, differentiation degree and complications. PIVKA-II expression obviously decreased after surgical resection.

**Conclusions:**

PIVKA-II is a promising serum biomarker for the HCC diagnosis that can be used as a supplement for AFP. The combined diagnosis of the two markers greatly improved the diagnostic efficiency of HCC. The PIVKA-II levels in HCC patients were widely associated with clinicopathological characteristics representing tumor cell dissemination and/or poor prognosis. PIVKA-II can be used to evaluate the curative effects of HCC resection.

## Background

Hepatocellular carcinoma (HCC) is one of the most common malignant tumors of the digestive system with high morbidity and mortality rates [1, 2]. Its early diagnosis is essential for timely treatment and improving survival [3]. Although ultrasound, magnetic resonance imaging (MRI) and other imaging techniques have greatly improved the accuracy of the HCC diagnosis, their application has been limited due to their disadvantages such as high cost, invasiveness and insensitivity to small tumors [4]. Therefore, the detection of convenient, inexpensive, non-invasive and repeatable serum biomarkers has played an important role in the HCC diagnosis [5, 6]. α-fetoprotein (AFP) is a biomarker widely used for the HCC diagnosis, but its diagnostic accuracy is limited since it has a high false-negative rate for detecting small and early-stage tumors. In addition, AFP may be elevated in some benign liver diseases, such as chronic hepatitis and cirrhosis without HCC [7]. At the moment, the AFP application for early screening of HCC has been controversial [8].

Therefore, it is essential to search new HCC-associated biomarkers, realize the combined detection of multiple indicators, improve the accuracy of early HCC diagnosis and reduce the missed diagnosis rate. Over the years, additional tumor markers for HCC have been suggested, such as Golgi protein 73 (GP73), Glypican-3 (GPC3), cytokeratin 19 (CK-19), among others [9–11]. GP73 has been considered a potential marker of HCC, but its serum levels in patients with hepatic parenchymal tumor may also be increased. Therefore, GP73 detection is not suitable to differentiate HCC and benign liver disease [9]. Liu et al. found that serum GPC3 levels were increased in HCC patients, however, GPC3 was not sensitive to the differentiation benign diseases from the early HCC [10]. Previous studies have shown that CK-19 expression is related to aggressive behavior in HCC, such as poor differentiated grade, metastasis and microvascular invasion, suggesting that CK-19 could be used as an indicator of the survival and recurrence in HCC patients [11]. However, these markers have not been considered effective enough for clinical application as indicators for HCC diagnosis.

In 1984, Liebman et al. found that prothrombin induced by vitamin K absence or antagonist-II (PIVKA-II) was significantly increased in the serum of HCC patients and it could serve as a new serum marker for HCC [12]. Some researchers believed that PIVKA-II was superior to AFP and could replace it in the diagnosis of HCC [13]. However, most studies have not reached this conclusion, they suggested that the combined detection of PIVKA-II and AFP may improve the HCC diagnosis compared to the use of each biomarker alone [14]. Therefore, the diagnostic value of PIVKA-II is controversial and it is still discussed whether there is a correlation between PIVKA-II and AFP and whether PIVKA-II can in fact completely replace or supplement the role of AFP in the HCC diagnosis [13, 14]. In addition, the understanding of the relationship between PIVKA-II and clinicopathological characteristics, as well as the role of PIVKA-II in assessing the HCC curative effects, have not been sufficiently investigated. These findings may contribute to a more comprehensive understanding of the significance of PIVKA-II in HCC.

Here, in order to solve these controversies, we detected serum PIVKA-II levels to evaluate the individual and combined diagnostic performances of PIVKA-II and AFP for HCC. The diagnostic ability of PIVKA-II for AFP-negative HCC was also evaluated. In addition, we analyzed the relationship between serum PIVKA-II levels and clinicopathological characteristics of HCC patients to study the value of PIVKA-II in assessing HCC progression and prognosis. Finally, we compared the serum PIVKA-II levels of 89 HCC patients before and after surgery to determine the role of PIVKA-II in evaluating the surgery curative effect on HCC.

## Methods

### Patients and specimens

This study analyzed a total of 471 serum samples obtained from 168 patients diagnosed with HCC, 150 patients diagnosed with benign liver disease and 153 healthy controls at Tianjin Medical University Cancer Institute and Hospital (Tianjin, China) between July 2017 and February 2019.

All procedures performed in this study that involved human participants were approved by the Research Ethics Committee of Tianjin Medical University Cancer Institute and Hospital and were in accordance with the ethical standards of the 1964 Helsinki Declaration. All subjects were over 18 years of age and signed the informed consent forms voluntarily.

All serum samples were collected before surgery or radiofrequency therapy and quickly frozen at − 80 °C until use, avoiding freezing and thawing cycles. Clinicopathological feature data including sex, age, tumor size and number, TNM stage, lymph node and distant metastasis, gross type, differentiation degree and complications (gastrointestinal bleeding, hepatic nephropathy, hepatic encephalopathy, rupture and hemorrhage of liver cancer nodule, secondary infection) were collected. Serum samples from 89 patients diagnosed with HCC were also collected 1 week after surgery. HCC was staged based on the American Joint Committee on Cancer (AJCC) TNM classification criteria (7th Edition).

All HCCs were diagnosed according to the “Diagnostic and Therapeutic Criteria for Primary Liver Cancer (2011 Edition)” issued by the Ministry of Health of China. Patients with hemorrhagic or thrombotic diseases (PTA < 40% or PT-INR ≥ 1.5) and those who have taken anticoagulant agents such as warfarin and similar drugs or vitamin K within 6 months of enrollment were excluded from this study.

### Serum analysis of PIVKA-II levels using enzyme-linked immunosorbent assays (ELISAs)

Serum PIVKA-II levels in HCC patients, benign liver disease patients and healthy controls were measured by ELISA (Eitest. Tokyo, Japan), according to the operation instructions provided by the manufacturer. Briefly, 100 μl diluent (blank), standard substances and serum samples were added in 96 well plates respectively. Then, the plates were incubated at 2–10 °C for 16–24 h. After three washes with PBST, 100 μl enzyme-labeled antibody was added and plates were incubated at 20–30 °C for 1 h. Subsequently, after three more washes with PBST, a total of 100 μl substrate solution and 50 μl stop solution were added to each well. The absorbance at 450 nm of blank, standard substances and samples were measured using a microplate reader (Thermo, Walsham, Massachusetts, USA). Each sample was measured in duplicate.

### Quantification of serum AFP levels using electrochemiluminescence immunoassays

Serum AFP levels were measured using the Roche Cobas E601 electrochemical immunoluminescence analyzer (Roche Diagnostics, Mannheim, Germany) equipped with Roche dedicated reagents following the instructions provided by the manufacturer.

### Statistical analysis

PIVKA-II and AFP values were expressed as median with an interquartile range. Wilcoxon signed-rank test was used to compare two groups and the Friedman rank sum test was used to compare three or more groups. Non-parametric Spearman’s rank correlation (r_s_) was employed to evaluate the relationship between serum levels of PIVKA-II and AFP in HCC patients. Receiver operating characteristic curve (ROC) analysis was performed to assess the diagnostic efficiency and obtain the area under the curve (AUC), cutoff values, sensitivity and specificity. AUCs were compared using Z tests. *P* values < 0.05 were considered statistically significant. All statistical analyses were performed using SPSS software version 17.0 (SPSS, Chicago, IL, USA).

## Results

### Elevated serum PIVKA-II levels in HCC patients

**Fig. 1:**
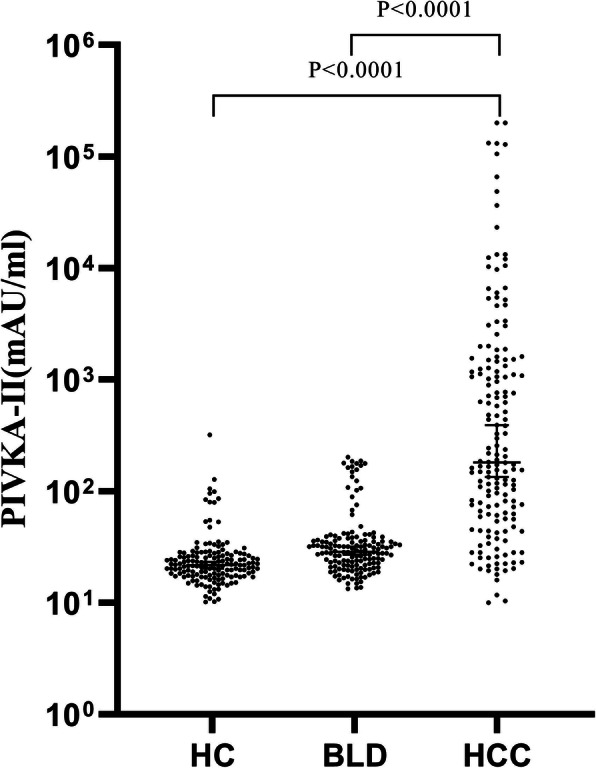
Expression levels of serum PIVKA-II in patients with HCC, benign liver diseases (BLD) and healthy controls (HC)

The ELISA analysis for PIVKA-II was performed on 168 HCC patients, 150 benign liver disease patients and 153 healthy controls. The serum concentrations of PIVKA-II in HCC patients was significantly higher than concentrations observed in patients diagnosed with benign liver disease and healthy controls (all *P* < 0.0001, Fig. 1). The median values for serum PIVKA-II amount in HCC, benign liver diseases and healthy controls were 181.50 (61.22–1314.00) mAU/ml, 28.60 (21.80–36.39) mAU/ml and 21.82 (17.65–26.53) mAU/ml, respectively. The results suggested that PIVKA-II can be used as a potential biomarker for HCC. In addition, there was no significant difference in the PIVKA-II levels between the benign liver disease patients and healthy controls.

### Diagnostic performances of PIVKA-II and AFP as individual biomarker for HCC

**Fig. 2:**
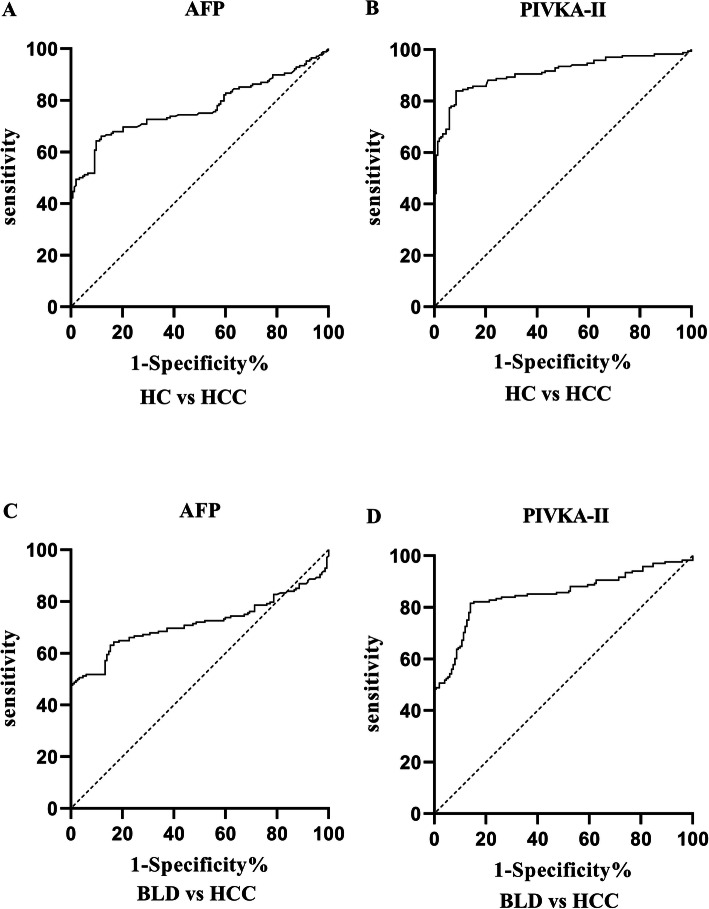
Performances of PIVKA-II and AFP in the diagnosis of HCC as individual biomarkers. ROC to distinguish HCC from healthy controls (HC) (**a**, **b**), HCC from benign liver diseases (BLD) (**c**, **d**)

A ROC curve was plotted to evaluate the performances of PIVKA-II and AFP as individual biomarker. As shown in Table 1 and Fig. 2, both markers showed remarkable diagnostic performance in distinguishing HCC from healthy controls (AUC _PIVKA-II_ = 0.90, 95%CI: 0.88–0.94; AUC_AFP_ = 0.77, 95%CI: 0.71–0.82). PIVKA-II was observed to have better diagnostic ability compared with AFP. Both markers showed similar performances when it came to distinguishing HCC from benign liver diseases (AUC _PIVKA-II_ = 0.85, 95%CI: 0.81–0.89; AUC_AFP_ = 0.72, 95%CI: 0.66–0.77).

**Table 1:** Performances of biomarkers for the diagnosis of HCC patients

	AUC(95%CI)	Cutoff value	Sensitivity(%)(95%CI)	Specificity(%)(95%CI)	Z test	*P* Value
**HCC vs Healthy controls**						
PIVKA-II	0.90 (0.88–0.94)	35.60mAU/ml	83.93 (77.63–88.71)	91.50 (86.01–94.97)		
AFP	0.77 (0.72–0.82)	17.76 ng/ml	64.29 (56.80–71.14)	90.20 (84.45–93.97)		
PIVKA-II + AFP	0.94 (0.92–0.97)^*^		87.50 (81.65–91.68)	92.50 (86.01–94.97)	6.756	< 0.01
**HCC vs Benign liver diseases**						
PIVKA-II	0.85 (0.81–0.89)	43.47mAU/ml	81.55 (75.00–86.68)	86.00 (79.54–90.66)		
AFP	0.72 (0.66–0.77)	21.47 ng/ml	63.10 (55.58–70.02)	84.67 (78.04–89.56)		
PIVKA-II + AFP	0.90 (0.87–0.94)^*^		81.95 (74.35–86.17)	89.33 (83.38–93.33)	6.744	< 0.01

^*^P < 0.01 in comparison with AFP

As shown in Table 1, the optimal cutoff values for PIVKA-II and AFP were obtained by Youden’s index. To discriminate HCC from healthy controls, the best cutoff value was 35.60mAU/ml for PIVKA-II, exhibiting a sensitivity of 83.93% and specificity of 91.50%. For AFP, the best cutoff value was 17.76 ng/ml with a sensitivity of 64.29% and specificity of 90.20%. To distinguish HCC from benign liver diseases, the best cutoff value was 43.47mAU/ml for PIVKA-II and the sensitivity and specificity were 81.55 and 86.00%, respectively. For AFP, the optimal cutoff value was 21.47 ng/ml and the sensitivity and specificity were 63.10 and 84.67%, respectively.

**Fig. 3:**
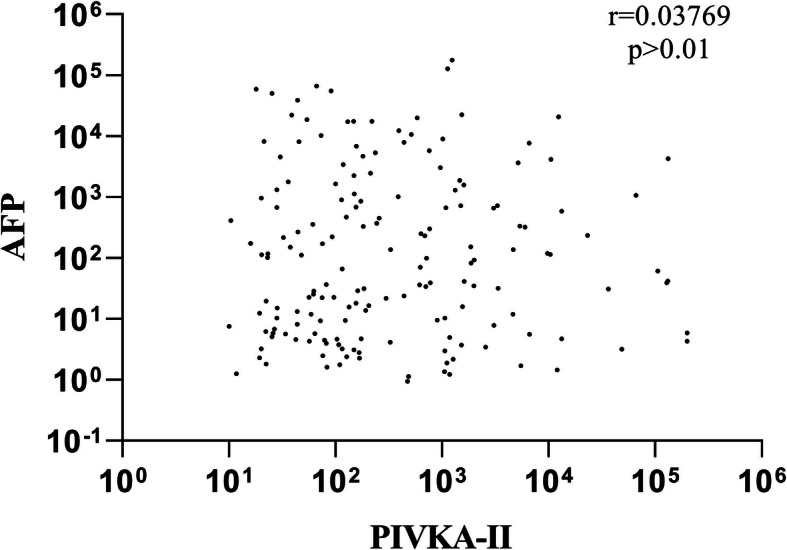
Correlation of serum PIVKA-II and AFP levels in HCC patients using the non-parametric Spearman’s rank correlation analysis

To assess the relationship between PIVKA-II and AFP in HCC, non-parametric Spearman’s rank correlation (r_s_) test was used. As illustrated in Fig. 3, no correlation was observed between serum levels of PIVKA-II and AFP in HCC (*r* = 0.03769, *P* > 0.01).

### Supplemental performance of PIVKA-II for AFP in the diagnosis of HCC

**Fig. 4:**
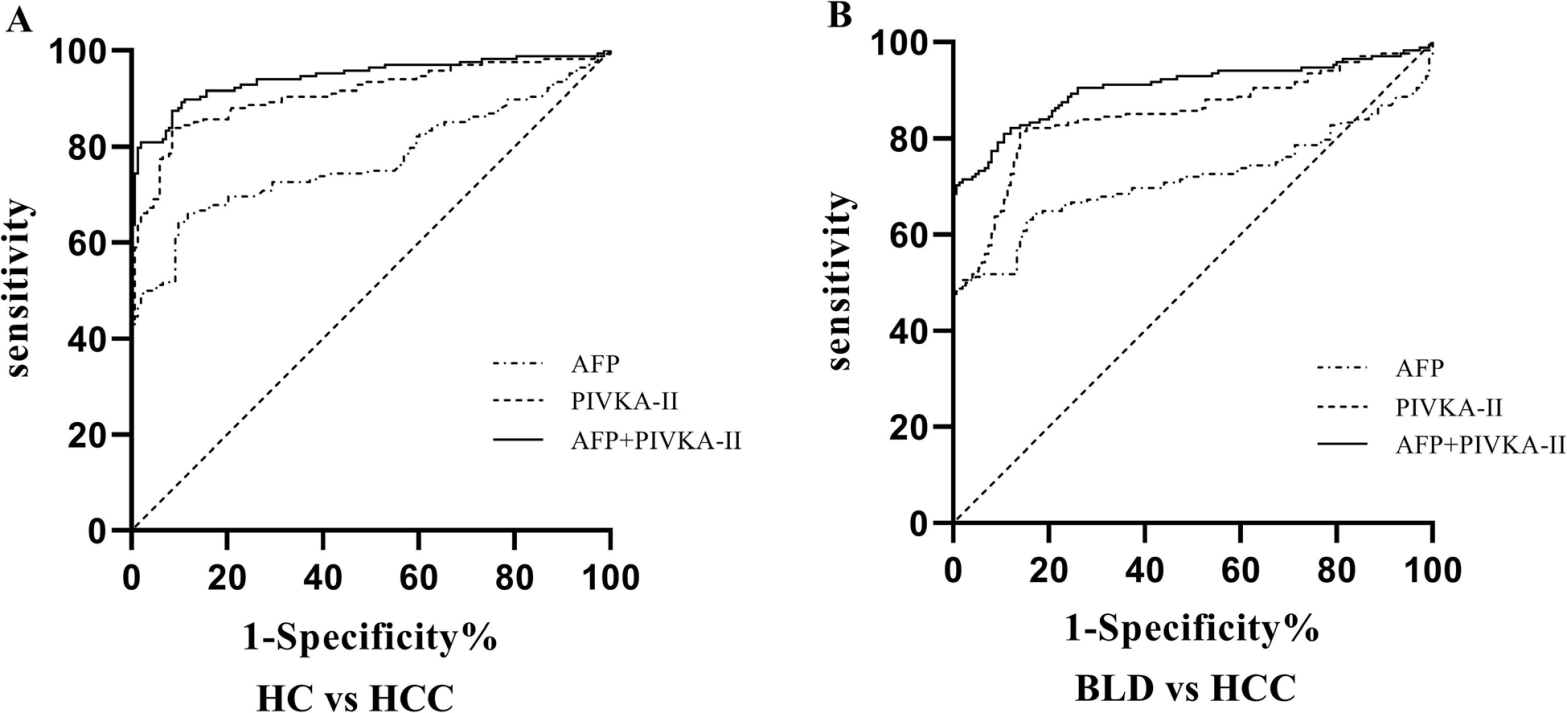
PIVKA-II and AFP complementation in the diagnosis of HCC. ROC of PIVKA-II, AFP, PIVKA-II + AFP to distinguish HCC from healthy controls (HC) (**a**), HCC from benign liver diseases (BLD) (**b**)

To estimate whether PIVKA-II improved the discrimination ability of AFP, the diagnostic efficacy of the two combined markers and AFP alone were compared. As shown in Fig. 4 and Table 1, the AUCs of the combined markers were significantly greater than AFP alone in all groups. To distinguish HCC from healthy controls, significant improvement was observed (AUC _PIVKA-II + AFP_ = 0.94 vs AUC_AFP_ = 0.77, z = 6.756, *P* < 0.01). The sensitivity and specificity of the diagnosis of the two combined markers increased to 87.5 and 92.5%, respectively. To discriminate HCC from benign liver diseases, the two markers combined were also more efficient than AFP alone (AUC _PIVKA-II + AFP_ = 0.90 vs AUC_AFP_ = 0.72, z = 6.744, *P* < 0.01). The sensitivity and specificity of the two markers combined detection increased to 81.95 and 89.33%, respectively.

### Efficacy of PIVKA-II in the diagnosis of AFP-negative HCC patients

**Fig. 5:**
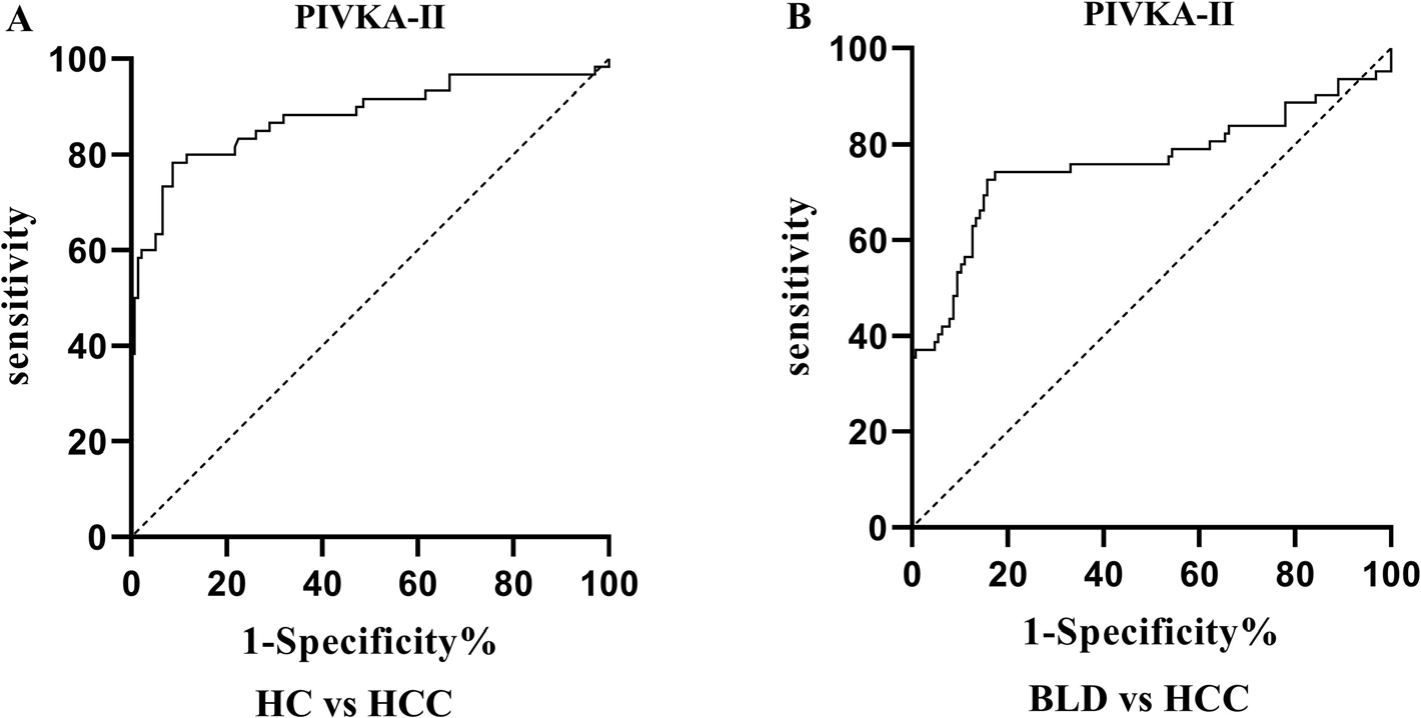
Performance of PIVKA-II in the diagnosis of AFP-negative HCC patients. ROC of PIVKA-II to distinguish HCC AFP-negative from healthy controls (HC) (**a**), and benign liver diseases (BLD) (**b**) respectively

To further explore the complementary role of PIVKA-II for AFP in the diagnosis of HCC, the diagnostic value of PIVKA-II were assessed in HCC patients that were missed by AFP, based on the cutoff values obtained in this study (17.76 ng/ml and 21.47 ng/ml respectively). As shown in Fig. 5 and Table 2, PIVKA-II showed a significant ability in distinguishing AFP-negative HCC from healthy controls (AUC_PIVKA-II_ = 0.88, *P* < 0.01) with a sensitivity of 78.33% and specificity of 91.3%, respectively. Moreover, the performance of PIVKA-II in discriminating AFP-negative HCC from benign liver diseases was still noticeable (AUC_PIVKA-II_ = 0.76, *P* < 0.01) with a sensitivity of 74.19% and specificity of 82.68%.

**Table 2:** Performance of PIVKA-II for the diagnosis of AFP-negative HCC patients

	AUC(95%CI)	Cutoff value	Sensitivity(%)(95%CI)	Specificity(%)(95%CI)
HCC vs Healthy controls	0.88 (0.82–0.94)	38.75mAU/ml	78.33 (66.38–86.88)	91.3 (85.42–94.96)
HCC vs Benign liver diseases	0.76 (0.67–0.85)	42.07 ng/ml	74.19 (62.12–83.45)	82.68 (75.16–88.27)

### Association between PIVKA-II and clinicopathological features in HCC

The relationship between serum PIVKA-II levels and clinicopathological features in HCC patients is summarized in Table 3. A significant correlation was observed between serum PIVKA-II concentrations and tumor size (*P* < 0.01). Moreover, serum PIVKA-II levels was positively correlated with the TNM stage (*P* < 0.01). Serum PIVKA-II levels in HCC patients with lymph node metastasis and distant metastasis were significantly higher than that observed in HCC patients without lymph node or distant metastasis (*P* < 0.01). The increased serum PIVKA-II values were related to a decrease in tumor cell differentiation (*P* < 0.01). Serum PIVKA-II levels in patients experiencing complications were significantly higher than the levels observed in patients without complications (*P* < 0.01). However, there was no significant correlation between the PIVKA-II abundance and age (*P* = 0.7477), sex (*P* = 0.6755), tumor number (*P* = 0.0143) or gross type (*P* = 0.6514).

**Table 3:** Correlation between serum PIVKA-II levels and clinicopathological characteristics in HCC

Characteristics	N (%)	PIVKA-II (mAU/mL) Median (Interquartile range)	P value
**Age (year)**			
<55	70 (41.67)	290.90 (61.32–1474.00)	
>55	98 (58.33)	156.30 (59.87–1264.00)	0.7477
**Sex**			
Male	117 (69.64)	172.40 (61.50–1212.00)	
Female	51 (30.36)	243.60 (45.23–1559.00)	0.6755
**Tumor size**			
<5 cm	53 (31.55)	127.00 (52.28–355.90)	
>5 cm	115 (68.45)	440.60 (62.74–2000.00)	< 0.01
**Tumor number**			
Single	137 (81.55)	166.30 (46.55–1151.00)	
Multiple	31 (18.45)	515.00 (109.80–6623.00)	0.0143
**Tumor stage**			
I	48 (28.57)	62.41 (25.43–412.00)	
II	57 (33.93)	174.30 (73.43–1059.00)	
III	26 (15.48)	775.9 (159.79–2241.00)	
IV	37 (22.02)	1854.00 (255.40–10,438.00)	< 0.01
**Lymph node metastasis metastasis**			
Yes	42 (25.00)	722.30 (162.00–2759.00)	
No	126 (75.00)	147.40 (46.55–1086.00)	< 0.01
**Distant metastasis**			
Yes	36 (21.43)	1745.00 (558.30–10,342.00)	
No	132 (78.57)	128.50 (44.04–710.90)	< 0.01
**Gross type**			
Nodular	23 (13.69)	328.20 (62.74–1854.00)	
Massive	93 (55.36)	185.40 (68.05–1394.00)	
Diffuse	52 (30.95)	163.90 (44.90–1150.00)	0.6514
**Differentiation degrees**			
Well	42 (25.00)	60.91 (27.62–334.30)	
Moderate	64 (38.10)	173.30 (49.39–1534.00)	
Poor	62 (36.90)	565.10 (148.10–1886.00)	< 0.01
**Complications**			
Yes	45 (26.79)	713.40 (184.00–3232.00)	
No	123 (73.21)	117.70 (42.34–1056.00)	< 0.01

### Abilities of PIVKA-II and AFP in evaluating a therapeutic response

**Fig. 6:**
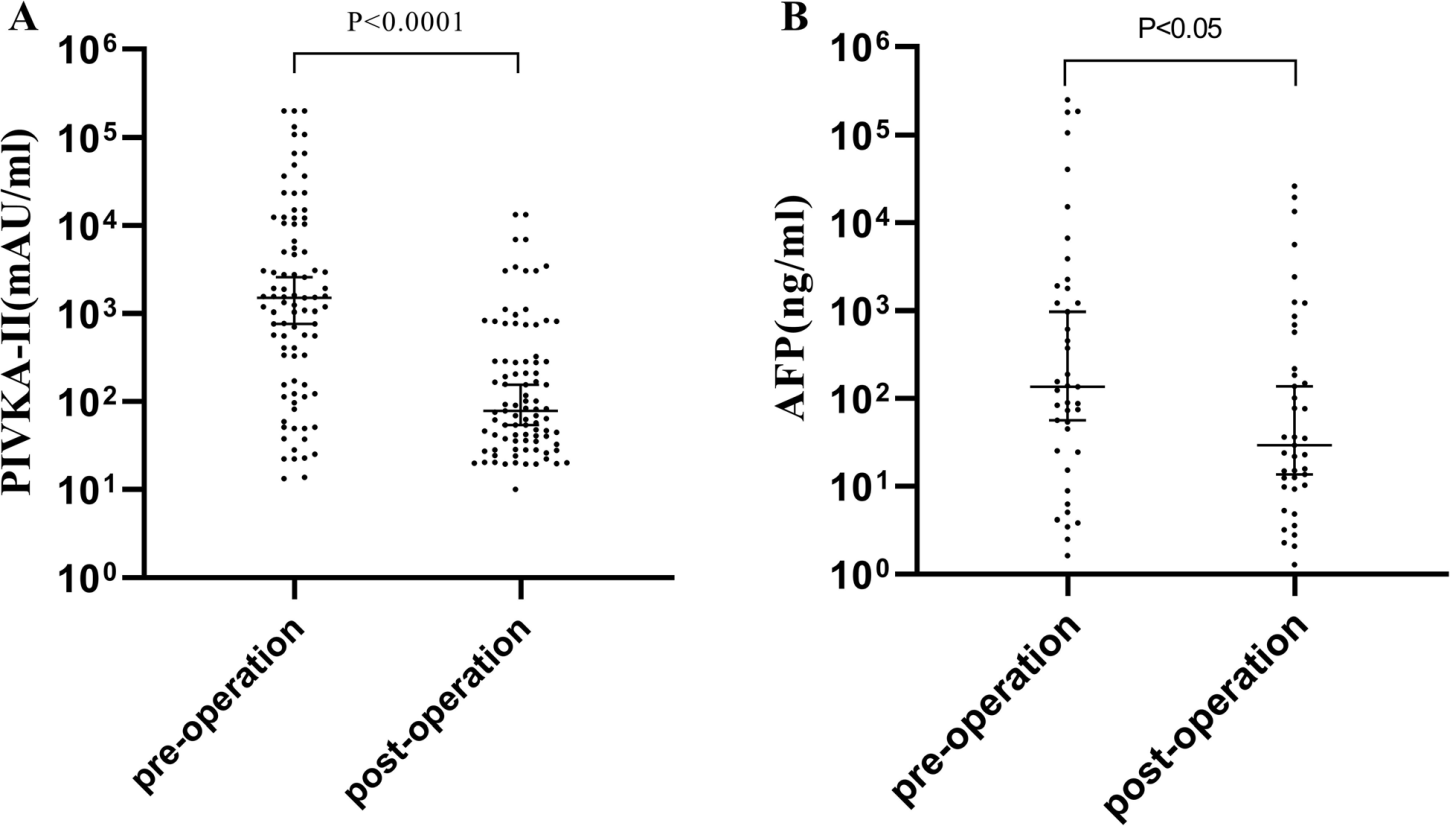
Tumor marker values before surgery and 1 week after surgery. **a**: PIVKA-II, **b**: AFP

A total of 89 paired pre- and post-operation serum samples obtained from HCC patients were analyzed and the PIVKA-II and AFP serum levels obviously decreased after surgery (Fig. 6). The serum PIVKA-II levels after surgery were 78.59 (35.50–287.60) mAU/ml and were significantly lower than that before surgery, where these levels measured 1498.00 (163.70–10,341.00) mAU/ml (*p* < 0.0001). AFP showed a similar trend. Serum AFP levels after surgery were 29.31 (10.04–392.90) ng/ml and were also significantly lower than that before surgery, where these levels measured 136.10 (24.84–1839.00) ng/ml (*p* < 0.05). PIVKA-II showed a more sensitive ability in evaluating therapeutic response of HCC resection than AFP.

## Discussion

HCC is a common malignant tumor of the digestive system, characterized by aggressive growth and early metastasis, and is the second leading cause of cancer mortality in China [1, 2]. As its early symptoms are not obvious, many HCC patients are diagnosed at an advanced stage [15]. Systematic screening of high-risk populations is necessary for early diagnosis. AFP is the most commonly used biomarker for HCC patients, despite its unsatisfactory sensitivity and specificity, especially for early-stage disease [7, 8]. As shown in the present study, the ability of AFP in diagnosis of HCC was relatively poor. The use of cutoff values of 17.76 ng/ml and 21.47 ng/ml would cause 60 (35.71%) and 62 (36.90%) of the 168 HCC patients to be considered negative, and 15 (9.80%) of 153 healthy controls and 23 (15.33%) of 150 benign liver disease patients would be considered positive. These inaccuracies support the inadequacy of AFP as a biomarker, as shown by previous work [16]. Therefore, novel and reliable biomarkers are needed to improve the HCC diagnostics.

Recently, the diagnostic role of PIVKA-II has been widely concerned. Normally, vitamin K is essential for the synthesis of coagulation factors II, VII, IX and X in the liver. In the absence of vitamin K or in the presence of antagonists, the activity of vitamin K-dependent carboxylase is inhibited, resulting in distubances in carboxylation of N-terminal glutamic acid residues of coagulation factors. This abnormal coagulation factor is unable to perform the clotting function and is known as prothrombin induced by vitamin K absence or antagonist-II (PIVKA-II) [12]. Previous studies have shown that PIVKA-II is a potential diagnostic marker for HCC [17, 18]. Our results showed that serum PIVKA-II levels in HCC patients were significantly higher than those observed in patients with benign liver diseases and healthy controls, the specific increase of PIVKA-II in HCC indicated that PIVKA-II may be a potential marker of HCC. These results were consistent with previous reports, [19, 20]the mechanism behind these effects have not been completely clear, however some researchers believed that it may be due to abnormal enzymes related to vitamin K metabolism generated during the malignant transformation of hepatocytes, which can lead to an increase of PIVKA-II levels [21].

The results of the present study showed that, as an individual biomarker, the performance of PIVKA-II was significant for the diagnosis of HCC. Moreover, the diagnostic ability of PIVKA-II was greater than that of AFP, as indicated by the diagnostic performance indicators, PIVKA-II showed higher AUC values and greater sensitivity and specificity than AFP. These conclusions were consistent with most previous studies [22]. The values of AUC, sensitivity and specificity of PIVKA-II in the diagnosis of HCC obtained from different studies were not exactly the same, which may be due to the different sample selection in different study designs. Although the diagnostic efficiency of PIVKA-II is better than that of AFP, we do not think that PIVKA-II can completely replace AFP for early screening of HCC. In comparison to the application of PIVKA-II alone, the combined detection of PIVKA-II and AFP was able to improve the accuracy of the HCC diagnosis. PIVKA-II may play an important complementary role for AFP, therefore a combined application of PIVKA-II and AFP is more desirable. In addition, unlike previous studies, we also evaluated the diagnostic value of PIVKA-II in the AFP-negative group, the study found that PIVKA-II showed moderate diagnostic ability for AFP-negative patients with HCC, which further proved the complementary role of PIVKA-II for AFP in the diagnosis of HCC. In conclusion, PIVKA-II can be considered a promising biomarker for the diagnosis of HCC.

Some researchers have studied the correlation between PIVKA-II and AFP in the HCC, most results have shown that there was no correlation between them, while a few researchers have concluded that there was a weak correlation between them [23]. The present study identified AFP and PIVKA-II were independent from each other and there was no correlation between them in the serum of HCC patients, which was consistent with most previous studies. This can be explained by different synthesis pathways of the two markers in hepatoma cells [24]. Thus, the application of these two complementary markers may be helpful for the diagnosis of HCC.

After confirming the PIVKA-II role in the HCC diagnosis, we attempted to explore the relationship between PIVKA-II and the progression and prognosis of HCC. In this study, we investigated these associations by analyzing clinicopathological characteristics including sex, age, tumor size and number, tumor stage, metastasis, gross classification, differentiation and complications of HCC patients. We found that serum PIVKA-II levels were positively correlated with the TNM stage and the tumor size, suggesting that PIVKA-II may play a role in predicting the severity of the disease. A higher concentration of PIVKA-II may suggest a larger tumor volume and a higher clinical stage. In addition, the PIVKA-II levels in HCC patients with lymph node metastasis and distant metastasis was significantly higher than that in patients without metastasis, Metastasis means poor prognosis, [25] therefore, the high concentration of PIVKA-II may reflect the poor prognosis of HCC patients to some extent. The differentiation degree refers to the proximity of cancer cells to normal cells, poorly differentiated tumors are more malignant, tend to grow rapidly and have a worse prognosis [26]. In this study, the PIVKA-II levels increased with the decrease of the differentiation degree of HCC, indicating that PIVKA-II has a certain relationship with the degree of malignant and the prognosis of HCC. Complications of HCC mainly occurred in the middle and late stages, are serious, fatal, and difficult to cure. It is one of the main causes of death of HCC [27]. Our study found that the serum PIVKA-II levels of patients with complications increased significantly, indicating that a high concentration of PIVKA-II may be associated with a poor prognosis. These results indicated that increased PIVKA-II concentrations may reflect a high degree of malignancy and poor prognosis of HCC. Long-term follow-up of HCC patients need to be conducted to reveal the potential role of serum PIVKA-II levels in the HCC prognosis.

Few previous studies on PIVKA-II have mentioned its role in assessing curative effect. By analyzing the changes of serum PIVKA-II levels in 89 HCC patients receiving surgical treatment, we found that the serum levels of PIVKA-II in HCC patients before and after surgery had a significant difference, suggesting that PIVKA-II may be used as an indicator in evaluating curative effects of liver cancer surgery. In addition, changes of PIVKA-II levels were more significant than those of AFP after surgery, which may be due to the shorter half-life of serum PIVKA-II (40–72 h) than that of AFP (5–7 days) [28]. These data suggest that PIVKA-II can reflect the curative effects of liver cancer surgery more timely.

A limitation of the present study is the lack of research on the relationship between PIVKA-II and survival and prognosis of HCC. Zhang et al. explored the relationship between PIVKA-II and the survival in HCC patients treated with curative ablation through meta-analysis. In that study, a total of 15 cohorts encompassing 5647 patients were included, and their results suggested that elevated PIVKA-II is a predictor of survival in HCC patients receiving curative ablation [29]. Surgery is the most effective method for the treatment of HCC, even in elderly patients [30]. Therefore, in the future, we should use longitudinal data to further explore the role of PIVKA-II in the prognosis and survival of HCC patients undergoing surgery.

## Conclusions

This study suggested that serum PIVKA-II levels may be an independent and useful tumor marker for the diagnosis of HCC. PIVKA-II was still valuable for the diagnosis of AFP negative HCC and can be used as a supplement of AFP in the diagnosis of HCC. The combined diagnosis of the two markers greatly improved the diagnostic efficiency of HCC. In addition, PIVKA-II values were associated with some pathological features that represent tumor invasiveness and/or poor prognosis. Finally, PIVKA-II can be used to evaluate the efficacy of liver cancer surgery.

## Data Availability

The datasets used during the current study are available from the corresponding author on reasonable request.

## Abbreviations

PIVKA-II: Prothrombin induced by vitamin K absence or antagonist-II
HCC: Hepatocellular carcinoma
AFP: α-fetoprotein
ROC: Receiver operating characteristic curve
MRI: Magnetic resonance imaging
GP73: Golgi protein 73
GPC3: Glypican-3
CK-19: Cytokeratin 19
AJCC: American Joint Committee on Cancer
ELISAs: Enzyme-linked immunosorbent assays
AUC: Area under the curve
